# In vitro propagation of *Codonopsis pilosula* (Franch.) Nannf. using apical shoot segments and phytochemical assessments of the maternal and regenerated plants

**DOI:** 10.1186/s12870-022-03950-w

**Published:** 2023-01-16

**Authors:** Roggers Gang, Richard Komakech, Yuseong Chung, Denis Okello, Wook Jin Kim, Byeong Cheol Moon, Nam-Hui Yim, Youngmin Kang

**Affiliations:** 1 Korean Convergence Medical Science Major, University of Science and Technology (UST), Daejeon, 34113 South Korea; 2 Herbal Medicine Resources Research Center, Korea Institute of Oriental Medicine (KIOM), 111 Geonjae-Ro, Naju-Si, South Korea; 3 National Agricultural Research Organization (NARO), National Semi-Arid Resources Research Institute (NaSARRI), Soroti, Uganda; 4 Natural Chemotherapeutics Research Institute (NCRI), Ministry of Health, Kampala, Uganda; 5 Department of Biological Sciences, Kabale University, P.O Box 317, Kabale, Uganda; 6 Korean Medicine Application Center, Korea Institute of Oriental Medicine, 70 Cheomdan-Ro, Dong-Gu, Daegu, 41062 South Korea

**Keywords:** Plant tissue culture, Plant growth regulators, FT-NIR, Genetic fidelity, Phenolics, Flavonoids, GC–MS, Antioxidant activity

## Abstract

**Background:**

*Codonopsis pilosula* (Franch.) Nannf. is a medicinal plant traditionally used in China, Korea, and Japan to treat many diseases including poor gastrointestinal function, low immunity, gastric ulcers, and chronic gastritis. The increasing therapeutic and preventive use of *C. pilosula* has subsequently led to depletion of the natural populations of this species thus necessitating propagation of this important medicinal plant. Here, we developed an efficient and effective in vitro propagation protocol for *C. pilosula* using apical shoot segments. We tested various plant tissue culture media for the growth of *C. pilosula* and evaluated the effects of plant growth regulators on the shoot proliferation and rooting of regenerated *C. pilosula* plants. Furthermore, the tissues (roots and shoots) of maternal and in vitro-regenerated *C. pilosula* plants were subjected to Fourier-transform near-infrared (FT-NIR) spectrometry, Gas chromatography-mass spectrometry (GC–MS), and their total flavonoids, phenolics, and antioxidant capacity were determined and compared.

**Results:**

Full-strength Murashige and Skoog (MS) medium augmented with vitamins and benzylaminopurine (1.5 mg·L^−1^) regenerated the highest shoot number (12 ± 0.46) per explant. MS medium augmented with indole-3-acetic acid (1.0 mg·L^−1^) produced the highest root number (9 ± 0.89) and maximum root length (20.88 ± 1.48 mm) from regenerated *C. pilosula* shoots. The survival rate of in vitro*-*regenerated *C. pilosula* plants was 94.00% after acclimatization. The maternal and in vitro-regenerated *C. pilosula* plant tissues showed similar FT-NIR spectra, total phenolics, total flavonoids, phytochemical composition, and antioxidant activity. Randomly amplified polymorphic DNA (RAPD) test confirmed the genetic fidelity of regenerated *C. pilosula* plants.

**Conclusions:**

The proposed in vitro propagation protocol may be useful for the rapid mass multiplication and production of high quality *C. pilosula* as well as for germplasm preservation to ensure sustainable supply amidst the ever-increasing demand.

**Supplementary Information:**

The online version contains supplementary material available at 10.1186/s12870-022-03950-w.

## Background

*Codonopsis pilosula* (Franch.) Nannf. is a medicinal plant traditionally used in China, Korea, and Japan to treat many diseases [1]. The plant is native to Asia and mainly found in East, South, and Central Asia [2]. The diseases *C. pilosula* is traditionally used to treat include poor gastrointestinal function, low immunity, gastric ulcers, and chronic gastritis, among others [3, 4]. Accordingly, *C. pilosula* is a common substitute for the more expensive *Panax ginseng* [5]. Several workers have previously evaluated the pharmacological potential of *C. pilosula* namely antitumor, antidiabetic, antimicrobial, and antiulcer activities [3, 6–8]. Particularly, a pectic polysaccharide (CPP1b) isolated from *C. pilosula* root exhibited antitumor activity through time and dose-dependent cytotoxic effects in human lung adenocarcinoma A549 cells [8]. Moreover, the CP polysaccharide (CPPA) of *C. pilosula* inhibited the invasion and migration of human epithelial ovarian cancer HO-8910 cells and exerted anti-proliferative effects on tumor cells in vitro [7]. As per antidiabetic activity, *C. pilosula* reduced blood glucose level and inhibited serum aldose reductase activity in diabetic mice [6]. Furthermore, Yang et al. [9] demonstrated the antimicrobial activity of *C. pilosula* leaves and roots against some bacteria and yeast. Inulin-type fructan CP-A extracted from *C. pilosula* roots significantly reduced the mucosal ulcer index in rats, demonstrating its potential therapeutic efficacy against acute gastric ulcers [3]. In another study, polysaccharides from *C. pilosula* showed immunoregulatory effect by restoring the levels of Interferon gamma (IFN-γ), Interleukin-10 (IL-10), Interleukin-2 (IL-2), and serum Immunoglobulin G (IgG) in mice [10]. These bioactivities of *C. pilosula* are attributed to its major constituent secondary metabolites, such as saponins, polysaccharides, sesquiterpenes, alkaloids, and essential oils [2, 11, 12].

The increasing therapeutic and preventive use of *C. pilosula* has remarkably increased its price, subsequently depleting the natural populations of this species and necessitating propagation of this important medicinal plant [13]. Indeed, a larger percentage of *C. pilosula* roots could be produced through cultivation [14, 15]. However, lower seedling viability, longer cultivation time, and higher labor demand for raising seedlings represent the major challenges in the conventional propagation of *C. pilosula* [13]. In addition, conventional seed propagation yields low-quality crop due to the occurrence of seed-borne diseases and xenogamous free pollination among different varieties [14, 16]. Therefore, micropropagation may be a sustainable alternative for the large-scale production of high-quality *C. pilosula.* Different methods of micropropagation have been developed and used for the fast production of several medicinal plant species [17]. In the case of *C. pilosula*, indirect regeneration from calli and somatic embryos has been attempted [18]. Furthermore, indirect propagation from callus protoplasts [19], direct organogenesis from dormant buds [13], and regeneration and multiplication through axillary bud induction [14] have been achieved. However, in most of these studies, successful shoot regeneration were achieved indirectly via a preceding phase of callus development and this is strongly associated with compromising the genetic fidelity of regenerants with respect to the mother plant [20–22]. Additionally, previous in vitro propagation studies on *C. pilosula* lacked important aspects, such as plant growth medium tests, and assessed only a few cytokinins for their shoot proliferation effects. Moreover, in vitro regeneration requirements vary from one explant type to another [23] yet to the best of our knowledge, direct in vitro regeneration of *C. pilosula* from apical shoot segments is not reported in the literature at present.

To this end, the present study aimed to develop an efficient and effective in vitro propagation protocol for *C. pilosula* from apical shoot segments. Various plant tissue culture media were tested for the growth of *C. pilosula*. Moreover, the plant growth regulators (PGRs) effects on shoot proliferation and rooting of the regenerated *C. pilosula* plants were assessed. Based on the fact that plant in vitro propagation sometimes cause somaclonal variation [17], we tested the genetic fidelity of the regenerants using RAPD. In addition, the maternal and in vitro-regenerated *C. pilosula* plant tissues (roots and shoots) were subjected to FT-NIR spectrometry, quantification of total flavonoids and phenols along with GC–MS analysis to compare their phytochemical compositions. Finally, the antioxidant capacity of the roots and shoots of regenerated and maternal plants was assessed. Overall, the proposed protocol can facilitate rapid yet sustainable production and multiplication of high quality *C. pilosula* as well as allow germplasm preservation using apical shoot segments.

## Results

### Effects of culture media on *C. pilosula* shoot growth

**Fig. 1:**
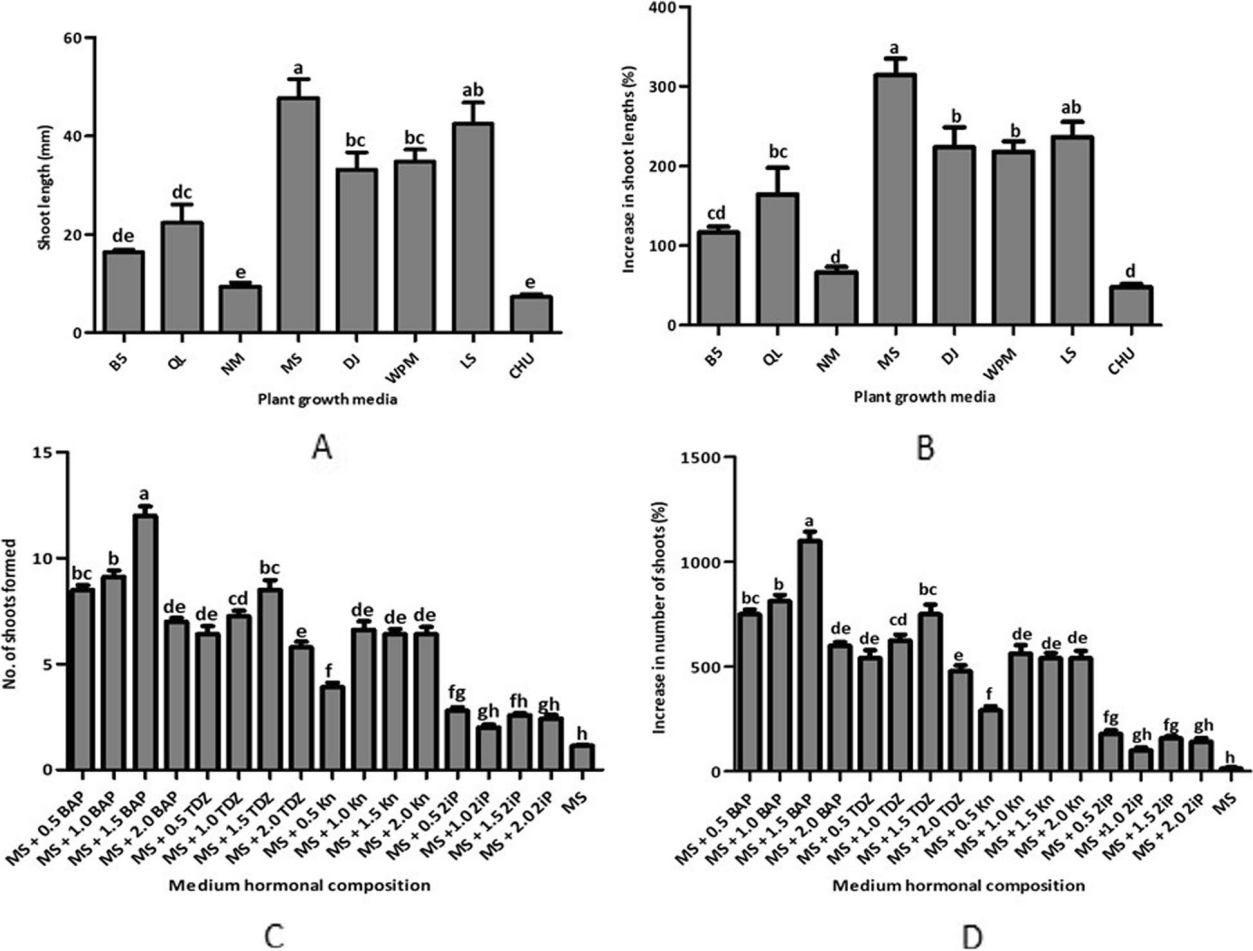
Plant growth media and cytokinins effects on *C. pilosula* growth and in vitro shoot regeneration from apical shoot explants, respectively. **A** Plant growth media effects on shoot lengths. **B** Plant growth media effects on percentage increase of shoot lengths. **C** Cytokinins effects on shoot number formed per apical shoot explant. **D** Cytokinins effects on percentage increase of shoot number per apical shoot explant. Same letters are not significantly different by Tukey’s test and *p* ≤ 0.05

Shoot growth of *C. pilosula* occurred in all tested basal media namely Linsmaier and Skoog (LS) medium, Quoirin and Lepoivre (QL) medium, Nitsch medium (NM), Murashige and Skoog (MS) medium, Gamborg’s B5 (B5) medium, Woody plant medium (WPM), De Greef & Jacobs (DJ) medium, and CHU medium after 6 weeks (Fig. 1A and 1B). The highest growth rate was recorded in MS medium with shoot length percentage increase of 314.23 ± 20.63% (shoot length, 47.7 ± 3.86 mm), although without significant difference from growth in LS medium (shoot length, 42.45 ± 4.36 mm; shoot length percentage increase, 236.36 ± 19.21%) (Fig. 1A and 1B). We also noted that the shoot and plant leaves grown in MS medium were more green. The lowest growth rate of 48.26 ± 3.45% increase in shoot length was recorded in Chu’s medium (shoot length, 7.3 ± 0.58 mm) (Fig. 1A and 1B). Therefore, MS medium produced the strongest growth effect on *C. pilosula* apical shoot explants.

### Effects of cytokinins on *C. Ppilosula* shoot proliferation

The various concentrations of all cytokinins namely isopentenyl adenine (2iP), 6-benzylaminopurine (BAP), kinetin (KN), and thidiazuron (TDZ) in MS medium enhanced shoot formation in *C. pilosula* (Fig. 1C and 1D). However, apical shoot proliferation varied among the cytokinin types and concentrations. BAP, Kn, and TDZ produced markedly higher number of shoots (*P* < 0.05) than control (MS) at all concentrations (Fig. 1C). The highest shoot number (12 ± 0.46) reflecting percentage shoot number increase of 1100.00 ± 46.24% was recorded in MS medium augmented with 1.5 mg·L^−1^ BAP, and this shoot number as well as percentage shoot number increase were markedly higher (*P* < 0.05) than those in the rest of the treatments (Fig. 1C and 1D). The lowest number of shoots (1.13 ± 0.07) was recorded in the control (MS) representing 12.50 ± 6.90% increase in number of shoots (Fig. 1C and 1D). Thus, 1.5 mg·L^−1^ BAP was optimal for *C. pilosula* shoot formation from the apical shoot segments.

### Effects of auxins on *C. Pilosula* root initiation and growth

**Fig. 2:**
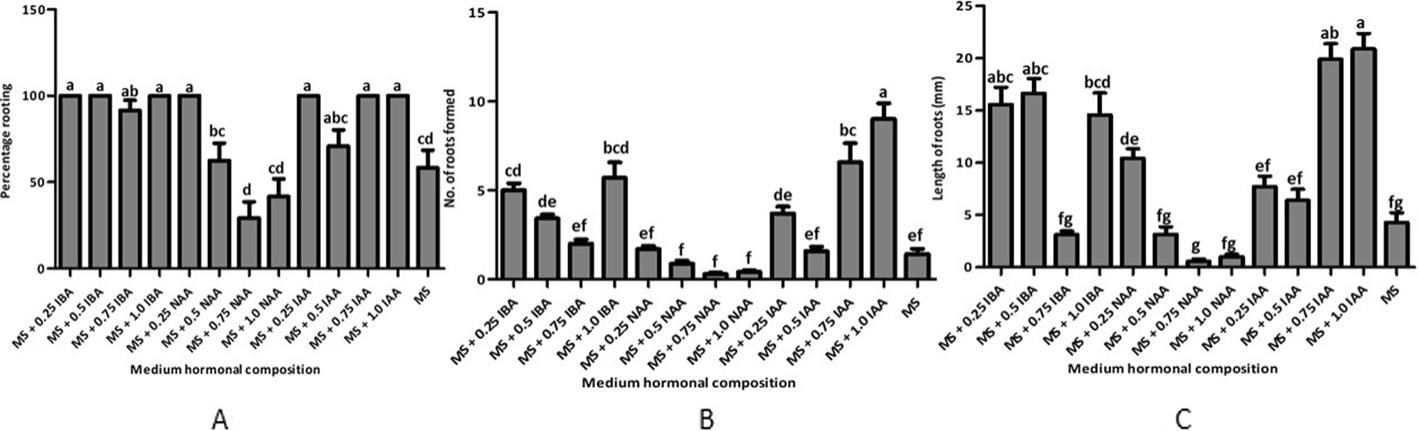
Effects of auxins on in vitro rooting of regenerated *C. pilosula* shoots in MS with vitamins fortified with 30% sucrose and different auxins at various concentrations. **A** Effects on percentage rooting. **B** Effects on number of roots formed per shoot. **C** Effects on root lengths. Same letters are not significantly different by Tukey’s test and *p* ≤ 0.05

*C. pilosula* roots were formed in all test media supplemented with auxins [indole-3-acetic acid (IAA), naphthaleneacetic acid (NAA), and indole-3-butyric acid (IBA)], albeit with significant differences in the number of roots, length of roots, and rooting percentage among the various rooting media (Fig. 2). Maximum percentage of rooting (100%) in regenerated shoots were recorded in media fortified with IBA (0.25, 0.50, and 1.00 mg·L^−1^), NAA (0.25 mg·L^−1^), and IAA (0.25, 0.75, and 1.00 mg·L^−1^); although these were not markedly (*P* < 0.05) higher than rooting percentages in media fortified with 0.75 mg·L^−1^ IBA and 0.50 mg·L^−1^ IAA. The lowest rooting percentage of 29.17% occurred in media fortified with 0.75 mg·L^−1^ NAA (Fig. 2A). Irrespective of the concentration, IAA produced the maximum root number, followed by IBA, while NAA formed the lowest root number (Fig. 2B). The maximum mean number of roots (9 ± 0.89) was registered in MS medium fortified with 1.0 mg·L^−1^ IAA, and this number was markedly higher than that in the rest of treatments. However, the lowest number of roots (0.29 ± 0.09) was observed in MS medium fortified with 0.75 mg·L^−1^ NAA (Fig. 2 B**)**.

The highest mean root length (20.88 ± 1.48 mm) was registered in MS medium augmented with 1.0 mg·L^−1^ IAA, although this number was not significantly higher than that recorded in MS medium augmented with 0.75 mg·L^−1^ IAA, 0.5 mg·L^−1^ IBA, or 0.25 mg·L^−1^ IBA (Fig. 2C**)**. The lowest mean root length (0.58 ± 0.2 mm) was registered in MS medium fortified with 0.75 mg·L^−1^ NAA (Fig. 2C**)**. Additionally, roots initiated in the medium fortified with IAA hormones were stronger and more firmly attached directly to the shoot base compared to those initiated in media fortified with NAA and IBA hormones, which were held loosely to the callus at the shoot base. Thus, MS medium augmented with 1.0 mg·L^−1^ IAA was optimal for root formation and growth in this experiment. The survival rate of acclimatized *C. pilosula* plantlets was 94.00% after 6 weeks. Thereafter, the in vitro-regenerated plants continued to grow vigorously without any observable difference in phenotype from the maternal plants.

### RAPD genetic fidelity assessment

**Fig. 3:**
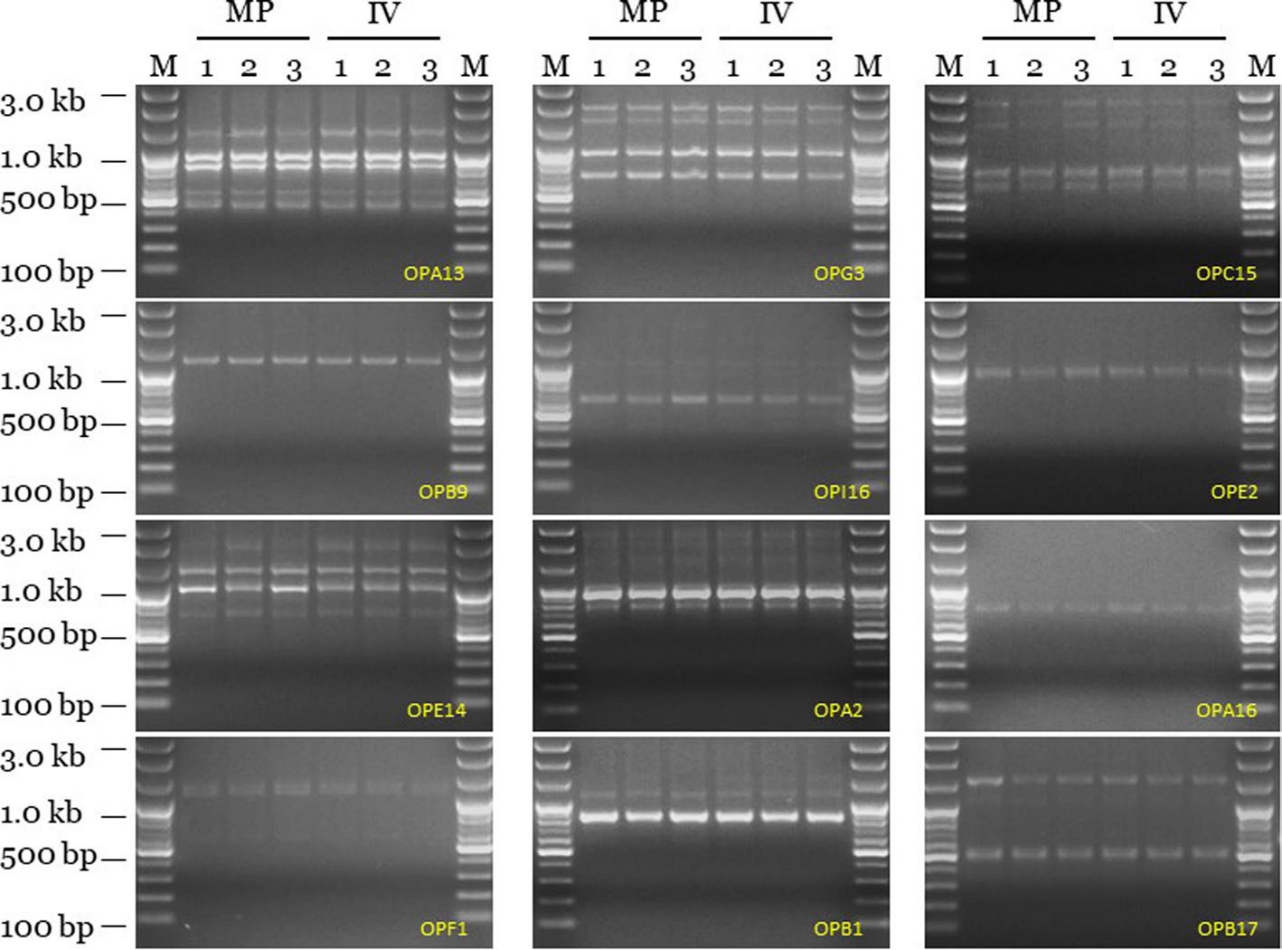
Randomly amplified polymorphic DNA Profiles regenerated by PCR amplification obtained with Operon primers. Lanes M-100 by plus DNA ladder, MP 1–3 *C. pilosula* maternal plant, IV 1–3 In vitro regenerated *C. pilosula* plants. Cropping is used for gels and blots in the main paper and full-length blots/gels are presented in Supplementary Fig. 1

Genetic fidelity analysis, using genomic deoxyribonucleic acid (DNA) from both the maternal *C. pilosula* plant (control) and the in vitro regenerated *C. pilosula* plants, was performed to determine genetic stability using RAPD markers. Twelve RAPD primers generated 29 scorable bands in numbers ranging from 500 to 3000 bp (Supplementary Table 1). The fingerprinting profiles of the *C. pilosula* plants using the RAPD markers showed distinct and reproducible amplified products (Fig. 3 and Supplementary Fig. 1 A, B, C, and D).

### FT-NIR spectra

**Fig. 4:**
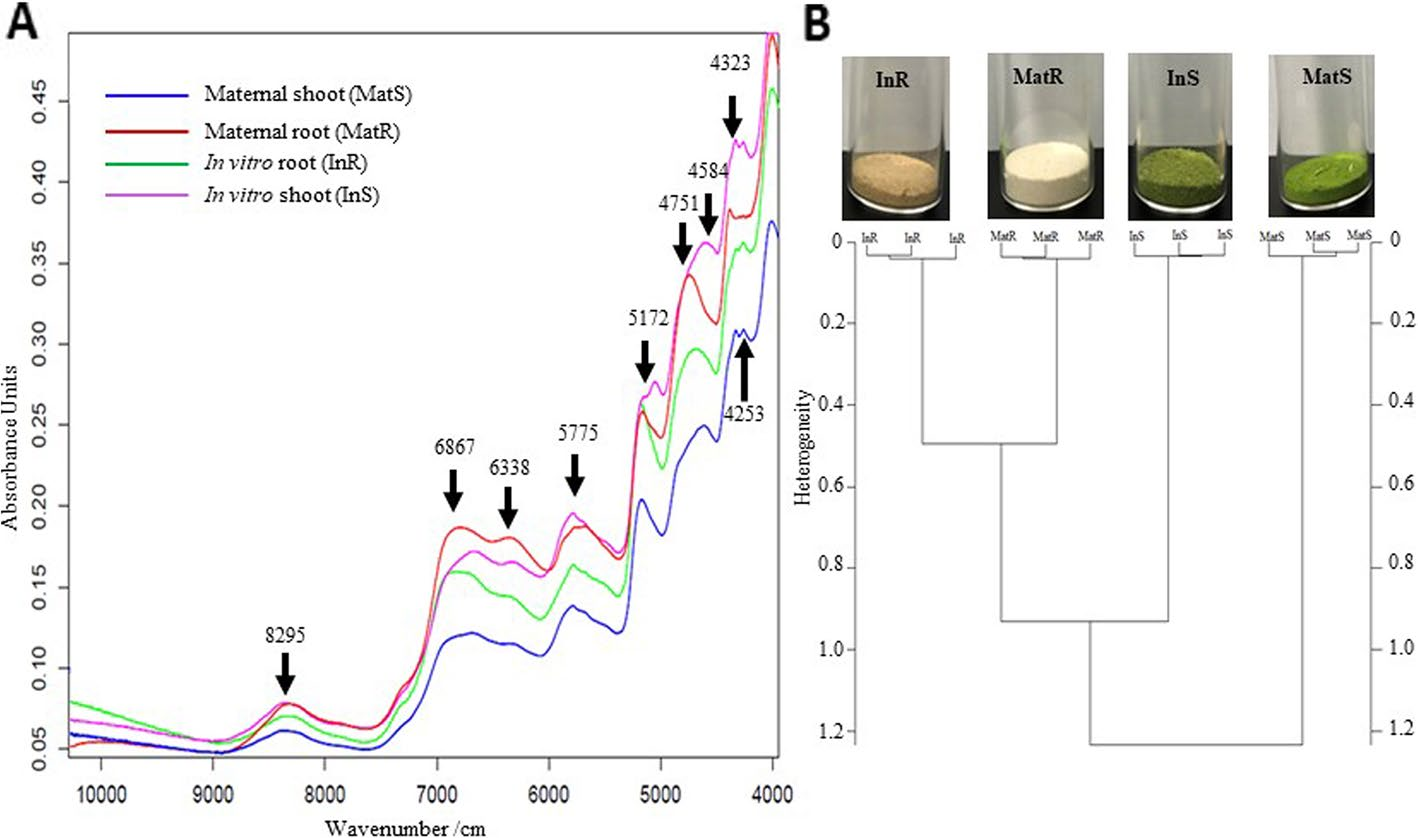
Characteristics of FT-NIR spectra between in vitro regenerated and maternal plants. **A** Comparison of FT-NIR spectral lines of samples from various parts of in vitro regenerated and maternal *C. pilosula*. **B** Clustering dendogram for different samples of in vitro regenerated and maternal *C. pilosula* plant analyzed from FT-NIR. In vitro regenerated *C. pilosula* plant samples analyzed: InR-root and InS-shoot. Maternal *C. pilosula* plant samples analyzed: MatR-root and MatS-shoot

The spectra of in vitro roots (InR), in vitro shoots (InS), maternal roots (MatR), and maternal shoots (MatS) were similar in general; however, between 5,000 and 6,000 cm^−1^ wavenumbers, the spectra of InS were slightly different (Fig. 4A). A peak was recorded at 4,751 cm^−1^ for InR and MatR and at 4,584 cm^−1^ for InS and MatS (Fig. 4A). Another peak was observed at 5,172 cm^−1^ for all samples, except InS (Fig. 4A). Furthermore, a peak was observed at 5,775 nm for all samples (Fig. 4A). Overall, seven prominent peaks were recorded in the FT-FNIR spectra of InR, InS, MatR, and MatS samples between 9,000 and 4,000 cm^−1^ wavenumbers (Fig. 4A). Ward’s algorithm-based clustering indicated an overall heterogeneity value of 1.27 among InR, InS, MatR, and MatS (Fig. 4B). InR plus MatR and InS showed a smaller heterogeneity value of 0.95 (Fig. 4B). The closest relationship was observed between InR and MatR, with a heterogeneity value of 0.5 (Fig. 4B).

### Antioxidant capacity of *C. Pilosula* tissues

**Fig. 5:**
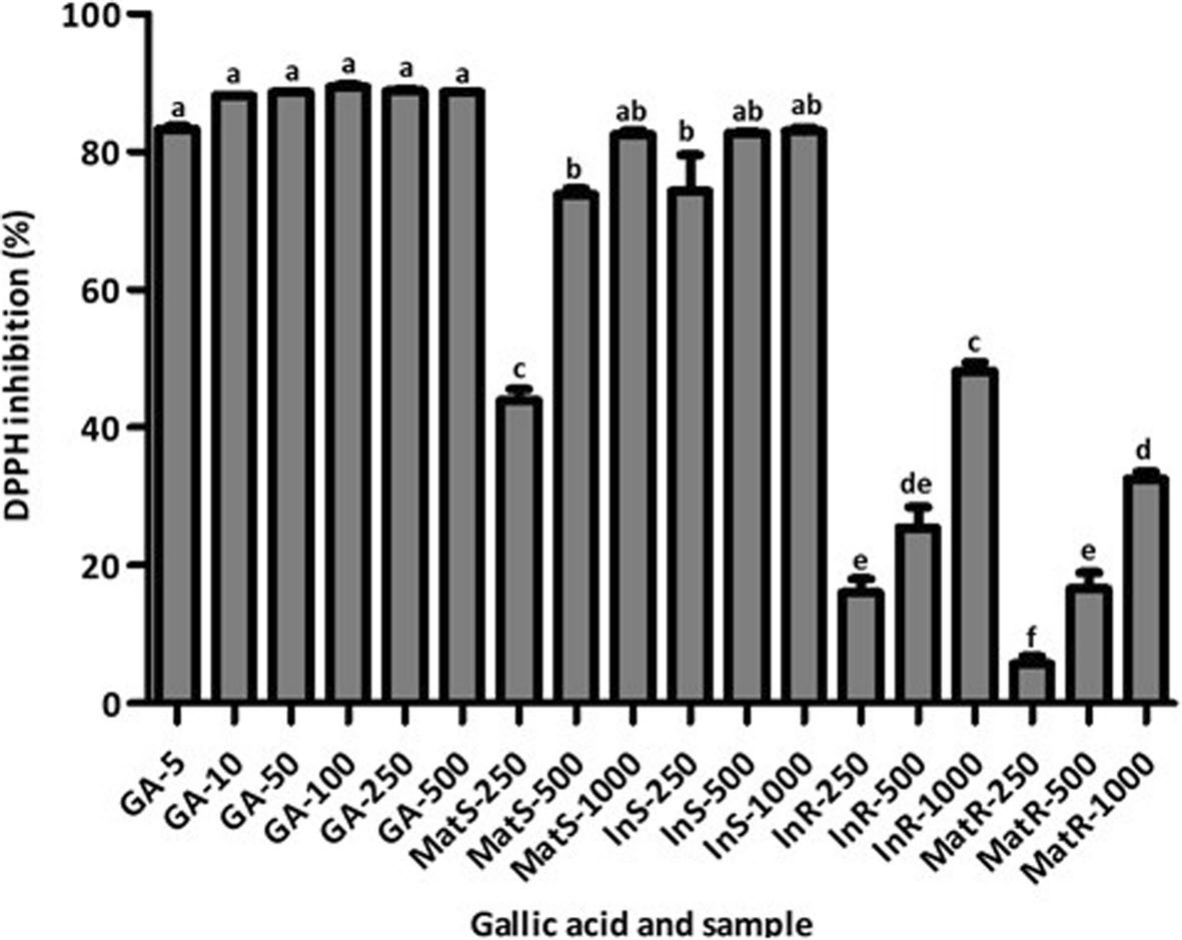
DPPH antioxidant activities of *C. pilosula* samples and gallic acid at different concentrations. Values are presented as means ± standard deviation. Same letters are not significantly different by Tukey’ s test and *p* ≤ 0.05

The diphenylpicrylhydrazyl (DPPH) antioxidant activity of *C. pilosula* samples varied according to sample type and concentration (Fig. 5). Generally, InS and MatS showed higher antioxidant activity than InR and MatR (Fig. 5). In all *C. pilosula* samples, the DPPH antioxidant activity increased with increasing concentration (Fig. 5); a pattern similar to that of positive control-gallic acid (GA) (Fig. 5). InS produced the highest DPPH free radical scavenging activity of 82.99 ± 0.096% and 82.71 ± 0.037% at concentrations of 1,000 and 500 mg·mL^−1^, respectively (Fig. 5). MatS produced the overall second highest DPPH free radical scavenging activity of 82.43 ± 0.110% and 73.76 ± 0.172% at the concentrations of 1,000 and 500 mg·mL^−1^, respectively (Fig. 5). The DPPH antioxidant activities of InS and MatS at all concentrations, except for MatS at 250 mg·mL^−1^, did not differ significantly (Fig. 5). Meanwhile, MatR produced the lowest DPPH antioxidant activity of 5.68 ± 0.797% at 250 mg·mL^−1^ concentration (Fig. 5). The DPPH antioxidant activity of MatR at a concentration of 1,000 mg·mL^−1^ did not differ from that of InR at a concentration of 500 mg·mL^−1^; the DPPH antioxidant activity of InR at a concentration of 500 mg·mL^−1^ was similar to that of MatR and InR at concentrations of 500 and 250 mg·mL^−1^, respectively (Fig. 5).

### Total flavonoid and phenolic content

**Fig. 6:**
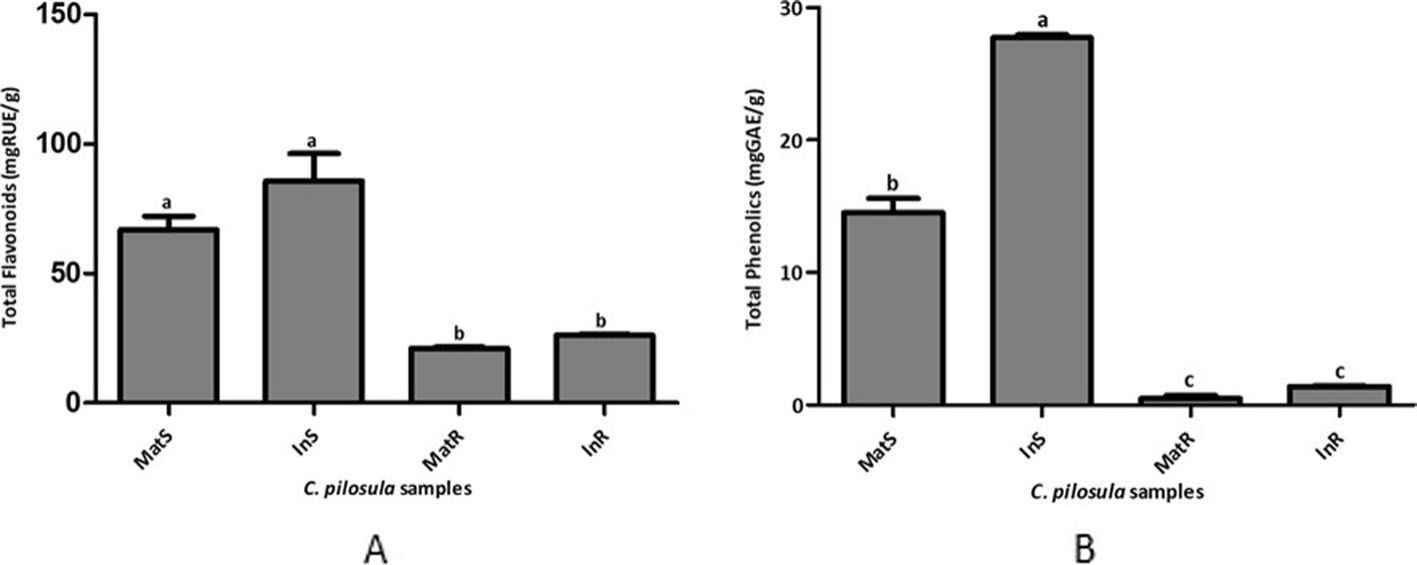
Total flavonoid and phenolic contents in *C. pilosula* samples. **A** Total flavonoid contents. **B** Total phenolic contents. Values are presented as means ± standard deviation. Same letters are not significantly different by Tukey’ s test and *p* ≤ 0.05

The total phenolic and flavonoid content markedly differed according to sample type (Fig. 6). InS and MatS showed significantly higher total phenolic and flavonoid contents than InR and MatR (Fig. 6). The highest total flavonoid content of 85.64 ± 1.994 mg RUE·g^−1^ was recorded in InS, although this value was not significantly different from that in MatS (Fig. 6A). Similarly, the highest total phenolic content of 27.72 ± 0.073 mg GAE·g^−1^ was observed in InS, and this value was markedly higher than that in other tested samples (Fig. 6B). Meanwhile, the lowest total flavonoid (21.03 ± 0.187 mg RUE·g^−1^) and phenolic (0.49 ± 0.530 mg GAE·g^−1^) content was recorded in MatR (Fig. 6). The values of total flavonoid and phenolic content in MatR did not significantly differ from those in InR (Fig. 6).

### Gas Chromatography-Mass Spectrometry (GC–MS) analysis of *C. pilosula* roots

The GC–MS chemical profiling of Maternal roots (MR) and In vitro roots (IR) extracts based on matching the mass spectra, retention times, and quality ratio analysis of libraries resulted into identification of 15 components from each extract (Supplementary Fig. 2 and Table 1). Among 15 components revealed in MR, the following showed the reliable mass as over 90% of quality: Cyclohexasiloxane (4), 2,4-Di-tert-butylphenol (5), (E)-2,6-Dimethoxy-4-(prop-1-en-1-yl) phenol (6), n-Hexadecanoic acid (7), n-Hexadecanoic acid, ethyl ester (8), (3aR,8aR,9aR)-8a-methyl-5-methylene-3-[(1-methylolpropylamino)methyl]-3a,4,4a,6,7,8,9,9a-octahydro-3H-benzo[f]benzofuran-2-one (9), (9Z,12Z)-octadeca-9,12-dienoic acid (10), Ethyl linolate (11), Hexadecanamide (12), 9-Octadecanamide (13), Erucylamide (14), and Vitamin E (15) (Supplementary Fig. 2A and Table 1). Among 16 components revealed in IR, the components that showed the reliable mass as over 90% of quality included 3,5-dihydroxy-6-methyl-2,3-dihydropyran-4-one (4), Cyclohexasiloxane (6), 2-Methoxy-4-vinylphenol (7), 2,6-dimethoxyphenol (8), 2,4-Di-tert-butylphenol (10), (E)-2,6-Dimethoxy-4-(prop-1-en-1-yl)phenol (11), n-Hexadecanoic acid (12), Hexadecanamide (13), 9-Octadecanamide (14), and Erucylamide (15) (Supplementary Fig. 2B and Table 1). The components that were only identified from MR included Normal-Hexadecanoic acid, ethyl ester, (3aR,8aR,9aR)-8a-methyl-5-methylene-3-[(1-methylolpropylamino)methyl]-3a,4,4a,6,7,8,9,9a-octahydro-3H-benzo[f]benzofuran-2-one, (9Z,12Z)-octadeca-9,12-dienoic acid, Linoleic acid, Vitamin E, and ethyl ester. Whereas 2-Methoxy-4-vinylphenol and 2,6-dimethoxyphenol were only identified from IR.

**Table 1:** Phytochemical components identified in the methanol extract of MR and IR by GC–MS analysis

Sample	Peak No	Identified Compound	*t*_*R*_ (min)	% of total	Quality (%)
**MR**	2	2,2-Dimethoxybutane	9.516	1.49	74
	3	3,5-dihydroxy-6-methyl-2,3-dihydropyran-4-one	23.310	0.76	83
	4	Cyclohexasiloxane	27.558	0.79	93
	5	2,4-Di-tert-butylphenol	33.320	1.13	97
	6	(E)-2,6-Dimethoxy-4-(prop-1-en-1-yl)phenol	37.932	1.06	95
	7	n-Hexadecanoic acid	43.192	4.51	99
	8	n-Hexadecanoic acid, ethyl ester	43.865	0.73	96
	9	(3aR,8aR,9aR)-8a-methyl-5-methylene-3-[(1-methylolpropylamino)methyl]-3a,4,4a,6,7,8,9,9a-octahydro-3H-benzo[f]benzofuran-2-one	45.839	0.66	93
	10	(9Z,12Z)-octadeca-9,12-dienoic acid	46.487	1.30	99
	11	Ethyl linolate	47.027	1.12	97
	12	Hexadecanamide	47.383	0.30	92
	13	9-Octadecanamide	50.531	4.81	97
	14	Erucylamide	57.031	8.97	99
	15	Vitamin E	64.452	1.78	91
	16	5-(4-Nitrophenyl)-1,3,4-oxadiazol-2(5H)-one	70.772	13.88	86
**IR**	2	Acetic acid ethyl ester	6.136	0.62	72
	3	2,2-Dimethoxybutane	9.516	0.64	74
	4	3,5-dihydroxy-6-methyl-2,3-dihydropyran-4-one	23.270	1.88	97
	5	4-Vinylphenol	25.430	0.88	87
	6	Cyclohexasiloxane	27.557	0.63	91
	7	2-Methoxy-4-vinylphenol	28.313	0.70	95
	8	2,6-dimethoxyphenol	29.255	0.49	97
	9	2-Cyclohexylpiperidine	29.957	0.79	72
	10	2,4-Di-tert-butylphenol	33.319	0.86	95
	11	(E)-2,6-Dimethoxy-4-(prop-1-en-1-yl)phenol	37.922	0.81	98
	12	n-Hexadecanoic acid	43.187	3.04	99
	13	Hexadecanamide	47.377	0.27	91
	14	9-Octadecanamide	50.529	2.60	98
	15	Erucylamide	57.029	4.63	99
	16	5-(4-Nitrophenyl)-1,3,4-oxadiazol-2(5H)-one	69.718	2.81	86

## Discussion

### Effects of culture media, cytokinins, and auxins on *C. Pilosula* shoot and root growth

During in vitro propagation, culture medium is the source of water and nutrients for plants; thus, plant growth and development depend on the medium [16, 17]. The growth and morphogenesis of plant tissues are controlled by the culture medium composition [23]. Given their different formulations (Table 2), plant media produce different growth effects [16, 17]. In general, the culture media vary in terms of the contents of plant macronutrients, such as ammonium nitrate [16]. For example, MS and LS contain higher ammonium nitrate of 1650.0 mg·L^−1^ compared to other media (Table 2). Notably, the optimum nutrient concentration requirement differs across species [23]. In the present study, MS medium optimally supported the nutrient requirements for *C. pilosula* growth. The more green leaves and shoots observed in MS grown plants could be associated with high content of nitrogen (N) in the medium due to the fact that N is key for chlorophyll synthesis, photosynthetic efficiency, and a contributor of approximately 41% of plant growth [24]. Successful regenerations of *C. pilosula* using MS medium were also reported previously [13, 14, 19].

**Table 2:** Compositions of the different plant tissue culture media used in this study

**Component**	**MS** [23]	**B5**[23]	**WPM**[23]	**NM**[23]	**LS** [25]	**QL** [26]	**DJ**[27]	**CHU**[28]
Ammonium nitrate	1650.0	-	400.0	720.0	1650.0	400	-	-
Ammonium sulphate	-	134.0	-	-	-	-	400.0	463.0
Boric acid	6.2	3.0	6.2	10.0	6.2	6.2	10.62	1.6
Calcium nitrate	-	-	386.0	-	-	-	-	-
Calcium chloride.2H_2_O	-	150.0	-	-	440.0	-	300.0	-
Calcium chloride, anhydrous	332.2	-	72.5	-	-	1200.0	-	125.34
Cobalt chloride•6H_2_O	0.025	0.025	-	-	0.025	0.025	0.0025	-
Cupric sulphate•5H_2_O	0.025	0.025	0.25	0.025	0.025	0.025	0.0025	-
Na_2_-EDTA	37.26	37.3	37.3	37.25	37.26	-	37.30	37.3
Sodium phosphate monobasic	-	130.42	-	-	-	-	250.0	-
Ferrous sulphate•7H_2_O	27.8	27.8	27.85	27.85	27.86	-	27.8	27.8
Magnesium sulphate	180.7	122.09	180.7	90.34	370.0	360.0	244.17	90.37
Manganese sulphate•H_2_O	16.9	10.0	22.3	18.94	22.3	1.0	1.68	3.33
Molybdic acid, sodium salt, 2H_2_O	0.25	0.25	0.25	0.25	0.25	0.25	0.0025	-
Potassium iodide	0.83	0.75	-	-	0.83	0.08	1.58	0.80
Potassium nitrate	1900.0	2500.0	-	950.0	1900.0	1800.0	2000.0	2830.0
Potassium sulphate	-	-	990.0	-	-	-	-	-
Potassium chloride	-	-	-	-	-	-	600.0	-
Potassium phosphate monobasic	170.0	-	170.0	68.0	170.0	270.0	-	400.0
Zinc sulphate•7H_2_O	8.6	2.0	8.6	10.0	8.6	8.6	1.06	1.5
Myo-inositol	100.0	100.0	100.0	100.0	100.0	-	100.0	-
Nicotinic acid	1.0	1.0	0.5	5.0	-	-	1.0	0.5
Pyridoxine HCl	1.0	1.0	0.5	0.50	-	-	1.0	0.5
Folic acid	-	-	-	0.50	-	-	-	-
Thiamine HCl	10.0	10.0	1.0	0.50	0.4	-	10.0	1.0
Glycine	-	-	2.0	2.0	-	-	-	2.0
Biotin	-	-	-	0.05	-	-	-	-

Every ingredient in mg·L^−1^

Cytokinins are plant hormones responsible for shoot formation and axillary shoot proliferation as well as elongation [17, 23]. Factors such as stability, conjugation rate, and oxidation, coupled with mobility, shape the differences in the ability of cytokinins to induce shoots in vitro [29]. Several plant tissues require a specific cytokinin for morphogenesis [23]. In the present study, 1.5 mg·L^−1^ BAP was optimal for *C. pilosula* shoot formation. Likewise, previous studies have reported the suitability of BAP for *C. pilosula* shoot formation [13, 14]. Furthermore, the best performance of BAP in either shoot formation or shoot proliferation of *Dianthus caryophyllus* L. [30], *Habenaria edgeworthii* Hook. f. ex. Collett [31], *Dioscorea deltoidei* Wall. ex Griseb. [32], and *Rhodiola imbricata* Edgew. [33] has been reported. The superiority of BAP in shoot formation over other cytokinins may be attributed to its ability to induce cell division and promote lateral bud development, which are key in breaking axillary bud dormancy [34, 35]. Furthermore, the greater stability of BAP in in vitro cultures has been implicated in its high shoot proliferative ability [29]. Decreased shoot formation at higher BAP concentrations may be attributed to vitrification [16, 29]. In a study by Slupski et al. [14], high BAP concentrations (20 μM) markedly reduced the survival rate of *C. pilosula*. Consistent with our finding, Vijendra et al. [36] have reported 1.5 mg·L^−1^ BAP as the optimal concentration for shoot formation (6.9 shoots per explant) in *Lucas aspera* Spreng. However, some studies have reported lower or higher optimal concentrations of BAP for shoot proliferation. For instance, 1.0 mg·L^−1^ BAP produced 2.90 shoots per explant on average in *Aloe vera* Linn. [37] but 19.50 shoots per explant on average in *Helianthemum germanicopolitanum* Bornm. [38]. Meanwhile, in *Boerhaavia diffusa* L., 2.0 mg·L^−1^ BAP produced the maximum mean number of shoot buds (6.65 per explant) [39]. Taken together, these reports imply that the variation in the optimum BAP concentration for shoot formation is linked to the type of explant and plant species [13, 40].

Auxins are important for root initiation, growth, and development [16]. In *C. pilosula*, root formation and growth were strongly affected by the type and concentration of auxins. Similarly, Zhang et al. [13] and Slupski et al. [14] reported that auxins enhanced rooting and root development from the regenerated shoots of *C. pilosula.* The observed differences in the number of roots formed, percentage rooting, and root growth among various auxins may be explained by the variability in their physiological activity and extent of movement through tissues (cell-bound or freely circulating) [23]. In the present study, 1.0 mg·L^−1^ IAA formed the highest mean number of roots (9 ± 0.89) and the maximum mean root length (20.88 ± 1.48 mm) in in vitro-regenerated *C. pilosula* shoots. The variation in the rooting of regenerated *C. pilosula* with the same hormone may be due to concentration-dependent cell elongation [41]. This result is consistent with that of Slupski et al. [14] who reported the highest rooting frequency with IAA in *C. pilosula.* The superiority of IAA in rooting has also been reported in many other plants, including *Stevia rebaudiana* (Bert.) [42] and *Aconitum ferox* Wall. ex Ser. [43]. In addition, many studies have reported the same concentration (1.0 mg·L^−1^) of IAA as optimal for rooting in various plants, including *Aegle marmelos* L. [44], *Rosa canina* L. [45], and *Stevia rebaudiana* Bertoni [46]. Contrary to our findings, however, several studies have noted maximum rooting at lower concentrations of IAA (e.g., 0.2 mg·L^−1^ IAA in *Stevia rebaudiana* Bertoni [47] and 0.5 mg·L^−1^ IAA in *Vanda pumila* Hook.f.) [48] or higher concentrations of IAA (e.g., 1.5 mg·L^−1^ IAA in *Prunus Africana*) [17]. These differences imply that the optimal auxin concentration to induce morphogenesis varies considerably among genera, species, and cultivars [23].

The acclimatization of in vitro-regenerated plants is critical for their survival under subsequent *ex vitro* conditions, because the potentially abnormal morphologies, physiologies, and anatomies of such plants warrant adaptation [16]. Indeed, in vitro plants transferred to *ex vitro* conditions gradually undergo leaf anatomical and morphological modifications, and their stomata begin functioning, as opposed to the constantly open stomata during in vitro growth [49]. In this study, the survival rate of in vitro*-*regenerated *C. pilosula* plantlets was 94.00%. Thus, the in vitro-regenerated plants were appropriately adapted to greenhouse conditions. The observed survival rate was comparable to that reported previously (90%) by Slupski et al. [14] in the same species.

### RAPD genetic fidelity assessment

Due to the fact that in vitro propagation of plants can potentially lead to genetic variation [50], it is essential to test genetic fidelity of the regenerants with respect to donor plant [51]. RAPD, a technique based on the non-coding regions of DNA, is one of the methods used for efficiently assessing genetic homogeneity and diversity [52]. As recorded in this study, the 29 amplified products were all monomorphic bands in the in vitro propagated plants in comparison to maternal *C. pilosula* plant. During the RAPD analysis of the In vitro propagated plants, no polymorphism was detected and this implies that there was genetical identity with maternal plant. Therefore, this result gives genetic information for in vitro propagated *C. pilosula* plants and revealed that the genetic fidelity of the micropropagated plants during the in vitro process was maintained. The observed genetic stability in this study may be attributed to meristem culture use (shoot tips), as organised meristems are generally resistant to genetic changes that could occur during cell division and in vitro differentiation [53, 54]. In plants, genetic stability and uniformity is important for proper growth, development, and reproduction [17]. Previously, in vitro propagated plants derived from shoot tips were reported to maintain genetic stability [53, 55]. Many workers have also used the RAPD marker technique to test variation in other plant species including, *Sapindus mukorossi* [56], *Rhynchostylis retusa* (L.) [57], *Thunbergia coccinea* Wall. ex D. Don [58], *Dendrobium moschatum* Sw*.* [59], and *Inula royleana* DC [60].

### FT-NIR spectra, antioxidant capacity, total flavonoid content, total phenolic content, and GC–MS analysis of *C. Pilosula* tissues

FT-NIR spectroscopy is a non-destructive phytochemical assay, and it has been extensively used to identify and characterize chemical compounds in samples [61]. FT-NIR spectra provide information on the major chemical bonds and chemical composition of samples [62]. The absorbance band at 8,273 cm^−1^ recorded in the present study resulted from the overtone of C-H stretch vibrations, which are associated with CH_2_ and CH_3_ groups [17]. The large absorbance peak recorded between 7,000 and 6,300 cm^−1^ originated from the overtones of O–H stretching, which is associated with starch, phenolic groups, carboxyl O–H groups, and water [63]. The absorbance peak observed at 5,775 cm^−1^ was attributed to the C-H stretching modes linked to aliphatic chains and aromatic rings. The sharp peak at 5,172 cm^−1^ was attributed to O–H stretching linked to water [16]. The peaks between 5,000 and 4,500 cm^−1^ resulted from C-H, N–H, and O–H stretching modes linked to proteins. The absorbance peak at 4,336 cm^−1^ was attributed to C-H stretching and ring deformation, while the peak at 4,253 cm^−1^ was attributed to the stretching of aliphatic and aromatic C-H [16]. The similar FT-NIR spectra of samples from in vitro-regenerated and maternal *C. pilosula* plants reflect homogeneity with respect to chemical composition. Furthermore, Ward’s algorithm was employed to cluster the shoot and root samples from maternal and in vitro-regenerated *C. pilosula* plants. This clustering method has been widely applied to a range of samples, including plants [17, 64]. In the present study, the smallest heterogeneity value (0.5) was observed between in vitro-regenerated and maternal roots (InR and MatR), indicating that InR and MatR shared a greater degree of homogeneity than InS and MatS. While the root InR plus MatR and InS showed a heterogeneity value of 0.95, the overall heterogeneity among InR, InS, MatR, and MatS was 1.27. In other words, the maternal and in vitro-regenerated *C. pilosula* parts were very similar, with close chemical phylogenetic relationships. The high level of homogeneity among the samples may be a result of similar chemical compositions revealed in the FT-NIR spectra of the samples. The small heterogeneity among the plant parts recorded may be due to the differences in the major compounds [16]. Similarly, Komakech et al. [17] and Okello et al. [16] have reported high homogeneity between the roots of in vitro-regenerated and maternal plants of *Prunus africana* (Hook.f.) Kalkman and *Aspilia africana* (Pers.) C. D. Adams, respectively. The small heterogeneity observed between the same plant parts of in vitro*-*regenerated and maternal plants may be attributed to age differences, since age affects the chemical composition of plants [65]. Similarly, many studies using Ward’s algorithm clustering have recorded heterogeneity in samples obtained from plants of different ages [16, 17, 66].

The DPPH radical scavenging assay has been widely used to determine the antioxidant activity of natural products [67]. This assay indicates the capacity of sample(s) to scavenge free radicals based on the constituent bioactive compounds [68]. Our DPPH free radical scavenging assay of *C. pilosula* samples showed that all tissues tested presented antioxidant properties. The antioxidant activity of *C. pilosula* tissues and isolates has been reported previously [3, 69–72]. Bioactive polyphenols and polysaccharides have been implicated in the antioxidant activity of plant tissues [70, 71, 73]. As such, the polyphenol content of plant samples is positively correlated with their antioxidant capacity [67, 68, 74]. Consistent with previous findings, the present study demonstrated that antioxidant activity was directly dependent on the total phenolic and flavonoid content of samples. Specifically, the high total phenolic and flavonoid contents resulted in high antioxidant activity. Among the tested samples, InS showed the highest total phenolic and flavonoid content, presenting the strongest DPPH free radical scavenging activity. In contrast, MatR showed the lowest total phenolic and flavonoid content, exhibiting the weakest DPPH free radical scavenging activity. Traditionally, it is the root of *C. pilosula* which is used for treatment of diseases [3, 4]. GC–MS phytochemical profiling of *C. pilosula* roots of in vitro regenerated plants (IR) and maternal plants (MR) revealed presence of several compounds; a total of 9 compounds out of 15 were identified from both MR and IR. However, some compounds were recorded only in either MR or IR. Age difference may be responsible for the observed variation because age has been previously reported to influence plant tissue chemical composition [75]. Variation in chemical composition of plant tissues due to age difference has also been registered in other species such as *Origanum vulgare* subsp. *gracile* [75] and *Nerium oleander* L. [76]. Indeed, some of the phytochemicals identified in IR and MR have been reported to exert antioxidant effects. Accordingly, 2,4-Di-tert-butylphenol exhibited marked antioxidant activity in 2,20 -azino-bis(3-ethylbenzthiazoline-6-sulfonic acid (ABTS) radical assay [77]. Yoon et al. [78] also reported that 2,4-Di-tert-butylphenol showed significant antioxidant activities in AAPH-mediated oxidation (IC_50_ = 9.9 µM), SIN-1-mediated oxidation (52%), and DPPH radical scavenging activity. In another study, 3,5-dihydroxy-6-methyl-2,3-dihydropyran-4-one was one of the major components of *Zingiber* officinale associated with strong antioxidant activity [79]. 2,2-Dimethoxybutane was the major compound in *Clematis graveolens* that showed antioxidant effects [80]. In light of these results, the antioxidant activity of *C. pilosula* tissues observed in the present study may be associated with their constituent polyphenols and other phytochemicals such as 2,4-Di-tert-butylphenol. Notably, the antioxidant activity and contents of total phenolic, total flavonoid, and other phytochemicals of in vitro-regenerated *C. pilosula* plant tissues were comparable to those of the same tissues of the maternal plant. Thus, in vitro regeneration did not alter the antioxidant capacity, total phenolic content, total flavonoid content, and phytochemical composition of *C. pilosula* plants. Similarly, Yang et al. [9] have reported higher antioxidant activity and total phenolic and flavonoid contents in the leaf tissues than in the root tissues of *C. pilosula.* Other workers have also identified similar phytochemicals in *C. pilosula* [81]. Meanwhile, Zhang et al. [82] noted differences in DPPH scavenging activity (aqueous extract = 40.9 ± 2.12 and ethanolic extract = 8.14 ± 0.51 ascorbate equivalent μM), total phenolic content (aqueous extract = 5.54 ± 1.36 and ethanolic extract = 3.78 ± 3.79 mg GAE·g^−1^), and total flavonoid content (aqueous extract = 4.09 ± 0.98 and ethanolic extract = 12.91 ± 0.98 mg·QE g^−1^) among different *C. pilosula* root tissue, which were attributed to the different solvents used for extraction.

## Conclusions

This study established an efficient, effective, and a reproducible in vitro propagation protocol for *C. pilosula* using apical shoot segments. Culturing apical shoot segments in full-strength MS medium including vitamins augmented with 1.5 mg·L^−1^ BAP optimally supported shoot regeneration. MS medium containing vitamins fortified with 1.0 mg·L^−1^ IAA was the most suitable for root initiation and elongation in in vitro-regenerated *C. pilosula* shoots. After acclimatization of the in vitro*-*regenerated *C. pilosula* plants to *exvitro* condition, their survival rate was 94.00%. The monomorphic bands observed using RAPD primers between the maternal plant and the in vitro regenerated plantlets, revealed genetic fidelity of the in vitro regenerated *C. pilosula* plants. Moreover, phytochemical composition of maternal and in vitro*-*regenerated *C. pilosula* plants determined by FT-NIR spectrometry, GC–MS, total phenolic and flavonoid content analysis were similar. Additionally, comparable antioxidant activities of in vitro-regenerated and maternal *C. pilosula* plant tissues were observed. Therefore, this protocol is suitable for the large-scale in vitro production of true to type and high-quality *C. pilosula* for medicinal use. Based on this, rapid and sustainable supply of *C. pilosula* for therapeutic purposes may be possible amidst the increasing demand. However, we recommend further studies on in vitro enhancement of secondary metabolite contents of *C. pilosula* as a basis for adding therapeutic value.

## Materials and methods

### Plant material and explants preparation

*C. pilosula* seeds were obtained from the Arboretum of Korea Expressway Corporation located at Jeonju city, Jeonbuk, Repulic of Korea (Supplementary Fig. 3A). Prior to seeds collection, the plant was identified by Dr. Sungyu Yang (Researcher at Korea Institute of Oriental Medicine (KIOM), Republic of South Korea). A voucher specimen (number KIOM202201023838) was deposited at the Korean Herbarium of Standard Herbal Resources (Index Herbarium code: KIOM) at KIOM, Herbal Medicine Resources Research Center, Republic of South Korea. *Codonopsis pilosula var. pilosula* was the variety of *C. pilosula* from which seeds were obtained and used. To obtain seedlings raised under aseptic conditions for use as explant excision sources in this experiment, the seeds were placed in washing test tubes and rinsed thrice for 5 min in succession with autoclaved double-distilled water. This was followed by surface sterilization with 70% ethanol (Daihan Scientific, Siheung, Korea) for 3 min and rinsing thrice with autoclaved double-distilled water. Thereafter, the seeds were surface sterilized for 2 min using 2% (v/w) sodium hypochlorite and immediately rinsed thrice with autoclaved double-distilled water. The seeds were then left to dry between sterile filter papers before inoculating on MS media supplemented with gibberellin (GA; 1.0 mg·L^−1^) (Supplementary Fig. 3B). Finally, the inoculated seeds were transferred to a culture room. Of note, every sterilization step was completed in a laminar flow clean bench. After germination and growth for 6 weeks, the apical shoot segments of the seedlings were used for the in vitro propagation experiment.

### Effects of culture media on *C. Pilosula* shoot growth

The following eight basal media were evaluated for their effects on apical shoot growth (Supplementary Fig. 3C and Table 2): LS medium, QL medium, NM, B5 medium, WPM, DJ medium, MS medium, and CHU medium. To each basal medium containing vitamins, 30 g·L^−1^ sucrose was added, and the pH adjusted to 5.7. Subsequently, 3 g·L^−1^ GELRITE (for solidification) was added, and the medium was autoclaved for approximately 20 min at 121 °C and then poured into 25 × 150 mm boiling tubes (borosilicate glass). Shoot apices were excised (20–25 mm), their initial length was measured, and they were singly inoculated into 50 mL of each of the above solidified basal medium in 25 × 150 mm boiling tubes (borosilicate glass). Twenty replicates were established (1 explant per tube × 8 media types × 20 replicates). The cultures were maintained under a 16 h photoperiod (33.73 µmol·m^−2·^s^−1^ light intensity) and 80% relative humidity. Shoot length data was collected from 20 plantlets per treatment after 6 weeks. The percentage increase in shoot length was then calculated using the formula:

Percentage increase in shoot length = $\frac{Final shoot length-Initial shoot length }{Initial shoot length}X 100$

The medium that showed the best results (MS) was used as the optimal growth medium for *C. pilosula* in subsequent experiments.

### Effects of cytokinins on *C. Pilosula* shoot proliferation

MS media with vitamins and 30 g·L^−1^ sucrose was fortified independently with four various cytokinins, namely 2iP, BAP, KN, and TDZ, at concentrations of 0.5, 1.0, 1.5, and 2.0 mg·L^−1^. pH was modified to 5.7. GELRITE (3 g·L^−1^) was added, and the mixtures were autoclaved for approximately 20 min at 121 °C. Then, 100 mL of the autoclaved medium was poured into a polystyrene culture vessel (125 × 110 mm, Gaooze 1011C culture vessel, Gyeonggi-do, South Korea) and allowed to cool and solidify. Apical shoots (length, 20 mm) were inoculated in MS media containing various cytokines (Supplementary Fig. 3D). Each culture vessel contained three apical shoots with eight replicates. After 6 weeks of growth, shoot number were recorded from 24 plantlets per treatment (Supplementary Fig. 3D1). The formula below was used to calculate the percentage increase in number of shoots:

Percentage increase in the number of shoots = $$\frac{Final number of shoots-Initial number of shoots}{Initial number of shoots}X 100$$

### Effects of auxins on *C. Pilosula* root initiation and growth

MS media containing 30 g·L^−1^ sucrose and fortified separately with three distinct auxins of IAA, NAA, and IBA at concentrations of 0.1, 0.25, 0.5, 0.75, and 1.0 mg·L^−1^ were prepared following the procedures used for preparing MS media supplemented with cytokinins. Regenerated apical shoots (length, 20 mm) were inoculated in triplicate into 100 mL of MS media supplemented with the three different auxins. Eight replicates were set for each treatment (3-regenerated apical shoot per culture vessel × 8 replicates). Root number, root length, and rooting rate ($$\frac{Number of shoots which formed roots in rooting medium}{Total number of plant shoots inoculated in the rooting medium}X 100$$) were recorded after 6 weeks from a total of 24 plantlets per treatment (Supplementary Fig. 3E).

### Acclimatization of regenerated *C. Pilosula* plants

After 6 weeks, regenerated *C. pilosula* plantlets were withdrawn with care from the culture vessels, growth medium was washed from the roots using sterile water, and planted in sterile horticultural soil blended with perlite (2:1) in plastic pots (13 × 11 cm) maintained in a greenhouse (Supplementary Fig. 3F). Plantlets were covered using transparent polythene bags for provision of optimum humidity, and the bags were progressively removed after 14 days. Irrigation was performed twice a week for the first 2 weeks and once a week for the following 4 weeks. The survival rate of plantlets was examined after 6 weeks of growth in the greenhouse.

### Genomic DNA isolation and RAPD analysis

RAPD analysis was performed on 7-month-old green house in vitro regenerated and maternal (control) *C. pilosula* plants in order to establish the genetic fidelity of the in vitro regenerated plants. Genomic DNA was extracted from fresh leaf tissue (100 mg) of both the in vitro regenerated plants (3) and the maternal plant acquired by randomly picking leaves from each set of plants using a DNeasy Plant Mini Kit (Qiagen, Germany). Prior to further analysis, purified DNA was stored at -20 °C. RAPD amplification was conducted in a reaction volume of 30 µL containing a 10 ng DNA template, 30 pmol of each random primer, and a Solg™ 2X Taq polymerase chain reaction (PCR) Smart-Mix I (Solgent, Daejeon, Korea). The amplification cycle comprised an initial denaturation step at 95 °C for 2 min, followed by 35 cycles of 1 min at 95 °C, 1 min at 42 °C, and 2 min at 72 °C, with a final extension step of 5 min at 72 °C. The amplification products were separated using a 1.5% agarose gel containing Eco Dye (Biofact, Daejeon, Korea). The sizes of the amplification products were obtained through comparison with a 100 bp DNA ladder (Solgent, Daejeon, Korea). The DNA bands in the gel were visualised under the Gel Doc System (Bio-Rad, Hercules, CA, USA) for photography and digitalisation of images.

### FT-NIR spectrometry

The roots and shoots of in vitro-regenerated (InR and InS, respectively) and maternal (MatR and MatS, respectively) plants were harvested. The plant parts were oven-dried for 48 h at 60 °C and subsequently pulverized to powder in a 250G pulverizer (model RT-N04-2 V, Taiwan) at 25,000 rpm. A TANGO FT-NIR spectrometer (Bruker Optics, Billerica, MA, United States) was used to analyze the powdered samples. Prior to the analysis, the spectrometer was calibrated using a light trap (type 1,002,961, ECL:00 and gold standard; type 1,024,957, ECL:01). Next, 3 g of each powdered sample was placed in vials (diameter, 20 mm) and analyzed. Classes of compounds in the samples with respect to functional groups were obtained between the absorbance spectra at 12,487 and 3,948 cm^−1^ wavenumbers. Sample dendrograms were created based on Ward’s algorithm clustering after characteristic data preprocessing (first derivative), vector normalization, and standardization of Euclidean distance in the 9,981–4,014 cm^−1^ frequency range. The algorithm was run in OPUS TANGO-R, and the minimum variance method was used to maximally sort homogeneous categories.

### Antioxidant assay

Harvested InR, InS, MatR, and MatS samples stored at 4 °C for 6 weeks were powdered. Next, 3 g of each powdered sample was added to 50 mL of 70% ethanol, followed by sonication for 1 h at 40 °C. The sample extracts were filtered (syringe filters with 0.45 µm pore size membranes), and the resulting filtrate was concentrated using a rotary evaporator (EYELA N-1200B, Tokyo Rikakikai Co. Ltd., Japan) under reduced pressure at 40 °C. Then, 10 mg of each concentrated *C. pilosula* sample was added to 5 mg of 70% ethanol to prepare a 2,000 μg·mL^−1^ stock solution. Thereafter, the stock solution for each sample was diluted to different concentrations (250, 500, and 1,000 μg·mL^−1^) for use in antioxidant assays.

To compare the antioxidant activity of different *C. pilosula* tissue samples, the diphenylpicrylhydrazyl (DPPH) free radical scavenging assay was conducted following the method of Okello et al. [68] with slight modifications. *C. pilosula* samples (100 µL) at different concentrations were added in triplicate to 100 µL of DPPH (Sigma-Aldrich, St. Louis, MO, USA) in ethanol in a 96-well microplate. The microplate was encased with aluminum foil and incubated for 30 min at 37 °C. Absorbance was measured on the Spectramax i3x spectrophotometer (Molecular Devices, Wokingham, UK) at 517 nm. The radical scavenging activity was calculated as percent antioxidant activity derived from the following formula:

Antioxidant activity (%) = $$\left[\frac{{{\varvec{A}}}_{{\varvec{c}}{\varvec{o}}{\varvec{n}}{\varvec{t}}{\varvec{r}}{\varvec{o}}{\varvec{l}}-{{\varvec{A}}}_{{\varvec{s}}{\varvec{a}}{\varvec{m}}{\varvec{p}}{\varvec{l}}{\varvec{e}}}}}{{{\varvec{A}}}_{{\varvec{c}}{\varvec{o}}{\varvec{n}}{\varvec{t}}{\varvec{r}}{\varvec{o}}{\varvec{l}}}}\right]\times 100$$

where *A*_*control*_ is the absorbance of the control sample and *A*_*sample*_ is the absorbance of the test sample. Gallic acid was the positive control.

### Total polyphenol content measurement

#### Total flavonoid content

To determine the total flavonoid content of *C. pilosula* samples (InR, InS, MatR, and MatS), the method described by Okello et al. [68] was adopted and slightly modified. Briefly, 100 µL of each sample extract (0.5 mg·mL^−1^) was added in triplicate to a 1.5 mL microcentrifuge tube, followed by the addition of 1 µL of diethyl glycol (90%). Next, 10 μL of 1 N sodium hydroxide solution was added to the constituents of each tube, and the mixture was vortexed for approximately 3 s and then incubated in a water bath for 60 min at 37 °C. Absorbance was measured on the Spectramax i3x (Molecular Devices) spectrophotometer at 420 nm. The total flavonoid content of each *C. pilosula* sample was obtained from a standard (rutin) calibration curve and expressed as milligram of rutin equivalent per gram of sample (mg RUE·g^−1^).

#### Total phenolic content

The total phenolic content was determined using a previously described method by Okello et al. [68] with some modifications. Briefly, 500 µL of 0.5 mg·mL^−1^ each sample was mixed with the same volume of Folin–Ciocalteu’s reagent in a 1.5 mL microcentrifuge tube in triplicate. After 3 min, 0.5 mL of 10% Na_2_CO_3_ was added to the mixture, and the solution was mixed thoroughly before incubating in the dark at 25 °C for 60 min. Absorbance was measured on the Spectramax i3x (Molecular Devices) spectrophotometer at 725 nm. The total phenolic content of each *C. pilosula* sample was obtained from a standard (gallic acid) calibration curve and expressed as milligram of gallic acid equivalent per gram of sample (mg GAE·g^−1^).

### Gas Chromatography-Mass Spectrometry (GC–MS) analysis of *C. Pilosula* roots

Roots for analysis were obtained from visibly healthy 8 weeks old in vitro regenerated plants and 4 month old maternal plants. The harvested roots were washed, oven dried for 48 h at 60 °C, homogenized into powder, and stored at 4 °C for 6 weeks prior to extraction. *C. pilosula* root powder of in vitro regenerated plants (IR) and root powder of maternal plants (MR) were extracted separately in 100% methanol by sonication for 30 min. Each extract was then prepared at 50 µg/mL; filtered through a 0.2 μm syringe membrane filter (Whatman Ltd, Maidstone, UK). Subsequently, the extracts were subjected to GC–MS analysis. Analysis was conducted in a 7890B GC–MS system (Agilent Technologies, Atlanta, GA, USA) along with a 7977B model mass detector (Agilent Technologies, Atlanta, GA, USA) utilizing a DB-5 MS capillary column (30 m × 0.25 mm × 0.25 μm). In brief, injection of 1 *μ*L extract in split mode at a ratio of 1/20 was done under chromatographic conditions with injection temperature and initial oven temperature of 250 °C and 50 °C, respectively. The initial oven temperature was then increased to 110 °C over the next 5 min and thereafter to 300 °C at a rate of 7 °C/min. The mass analyzer was set to scan from 30 to 600 amu. Peaks were distinguished through comparison with experimental mass spectra at the National Institute of Standards and Technology (NIST) and Wiley GC–MS libraries.

### Statistical analysis

One-way analysis of variance (ANOVA) was under taken to analyze all experimental data, followed by Tukey’s post hoc tests using GraphPad Prism v 5.03. Differences were considered significant at *P* < 0.05.

## Supplementary Information

**Additional file 1:** **SupplementaryFig. 1.** Full-length membranes of randomly amplified polymorphic DNA Profilesregenerated by PCR amplification obtained with Operon primers for maternal andIn vitro regenerated *C. pilosula* plants : (A) Operon primers OPA-13, OPB-9, andOPE-14. (B) Operon primers OPF-1, OPG-3, and OPI-16. (C) Operon primers OPA-2,OPB-1, OPC-15, and OPE-2. (D) Operon primers OPA-16 and OPB-17. Lanes M-100 byplus DNA ladder, MP 1-3 *C. pilosula* maternal plant, IV 1-3 In vitro regenerated *C. pilosula* plants. **Supplementary Fig. 2.** GC-MS chromatogram of the methanolextract of *C. pilosula *root samples. (A) GC-MS chromatogram of the *C. pilosula* roots of maternal plant (MR). (B) GC-MS chromatogram of the *C. pilosula* roots of in vitro regenerated plants (IR). **Supplementary Fig. 3.** *C. pilosula* in vitropropagation through apical shoot in optimum growth conditions. (A) Seed of *C.pilosula*. (B) In vitro *C. pilosula* seedling for use as explants. (C) Media testfor growth of *C. Pilosula* (D) Test of different cytokines for shootproliferation of *C. Pilosula.* (D1) Multiple shoots developing from a singleapical shoot. (E) Test for rooting of *C. pilosula* in different Auxins. (F)Acclimatized regenerated *C. pilosula* plant with well-developed root and shootsystems in horticulture soil mixed with perlite in the ratio of 2:1. **SupplementaryTable 1.** List of primers, their sequences, number of scorable bands andapproximate sizes of the amplified fragments generated by the 12 RAPD markers.

## Data Availability

The datasets used and/or analysed during the current study are available from the corresponding author on reasonable request.

## Abbreviations

FT-NIR: Fourier-transform near-infrared spectrometry
GC–MS: Gas chromatography-mass spectrometry
DNA: Deoxyribonucleic acid
RAPD: Randomly amplified polymorphic DNA
MS: Murashige and Skoog medium
LS: Linsmaier and Skoog medium
QL: Quoirin and Lepoivre medium
NM: Nitsch medium
B5: Gamborg’s B5 medium
WPM: Woody plant medium
DJ: De Greef & Jacobs medium
2iP: Isopentenyl adenine
BAP: 6-Benzylaminopurine
KN: Kinetin
TDZ: Thidiazuron
IAA: Indole-3-acetic acid
NAA: Naphthaleneacetic acid
IBA: Indole-3-butyric acid
PCR: Polymerase chain reaction
DPPH: Diphenylpicrylhydrazyl
