# Supplementary material for: In vitro propagation of Codonopsis pilosula (Franch.) Nannf. using apical shoot segments and phytochemical assessments of the maternal and regenerated plants
Source: BMC Plant Biol. 2023 Jan 16;23:33. doi: 10.1186/s12870-022-03950-w (PMC9841653; doi:10.1186/s12870-022-03950-w)
Supplement: Supplementary file 1 — Additional file 1: SupplementaryFig. 1. Full-length membranes of randomly amplified polymorphic DNA Profilesregenerated by PCR amplification obtained with Operon primers for maternal andIn vitro regenerated C. pilosula plants : (A) Operon primers OPA-13, OPB-9, andOPE-14. (B) Operon primers OPF-1, OPG-3, and OPI-16. (C) Operon primers OPA-2,OPB-1, OPC-15, and OPE-2. (D) Operon primers OPA-16 and OPB-17. Lanes M-100 byplus DNA ladder, MP 1-3 C. pilosula maternal plant, IV 1-3 In vitro regenerated C. pilosula plants. Supplementary Fig. 2. GC-MS chromatogram of the methanolextract of C. pilosula root samples. (A) GC-MS chromatogram of the C. pilosula roots of maternal plant (MR). (B) GC-MS chromatogram of the C. pilosula roots of in vitro regenerated plants (IR). Supplementary Fig. 3. C. pilosula in vitropropagation through apical shoot in optimum growth conditions. (A) Seed of C.pilosula. (B) In vitro C. pilosula seedling for use as explants. (C) Media testfor growth of C. Pilosula (D) Test of different cytokines for shootproliferation of C. Pilosula. (D1) Multiple shoots developing from a singleapical shoot. (E) Test for rooting of C. pilosula in different Auxins. (F)Acclimatized regenerated C. pilosula plant with well-developed root and shootsystems in horticulture soil mixed with perlite in the ratio of 2:1. SupplementaryTable 1. List of primers, their sequences, number of scorable bands andapproximate sizes of the amplified fragments generated by the 12 RAPD markers. [file 12870_2022_3950_MOESM1_ESM.pdf]

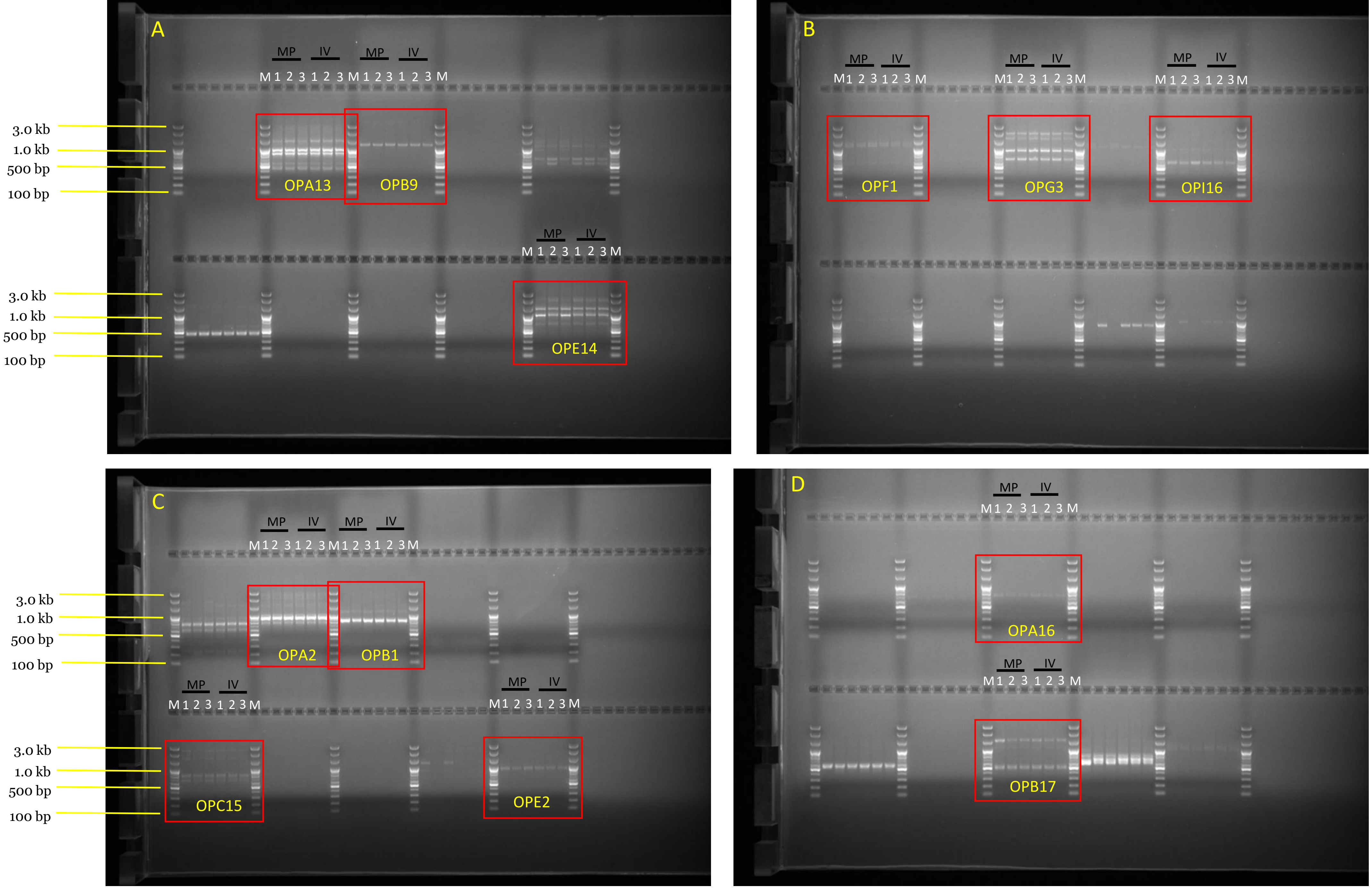

Supplementary Fig. 1 Full-length membranes of randomly amplified polymorphic DNA Profiles regenerated by PCR amplification obtained with Operon primers for maternal and *In vitro* regenerated *C. pilosula* plants : (A) Operon primers OPA-13, OPB-9, and OPE-14. (B) Operon primers OPF-1, OPG-3, and OPI-16. (C) Operon primers OPA-2, OPB-1, OPC-15, and OPE-2. (D) Operon primers OPA-16 and OPB-17. Lanes M-100 by plus DNA ladder, MP 1-3 *C. pilosula* maternal plant, IV 1-3 *In vitro* regenerated *C. pilosula* plants.

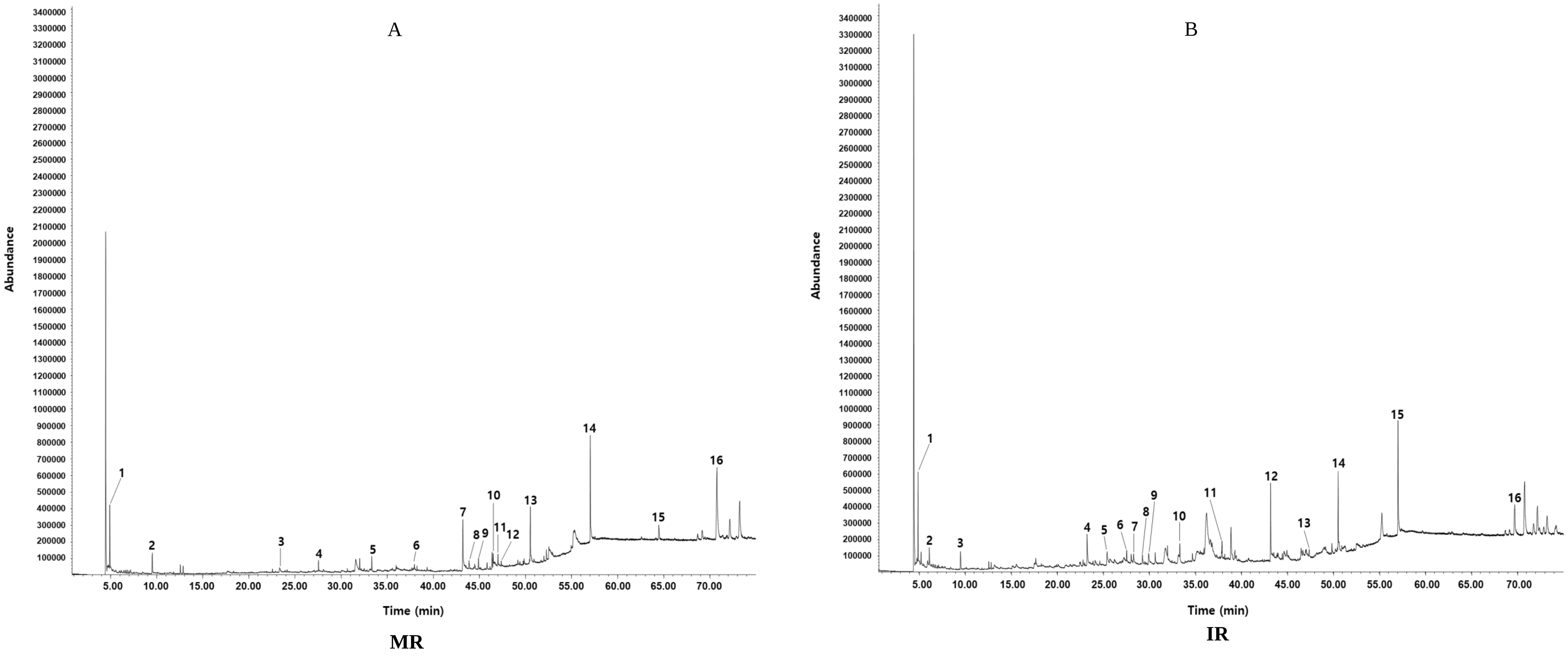

Supplementary Fig.2 GC-MS chromatogram of the methanol extract of *C. pilosula* root samples. (A) GC-MS chromatogram of the *C. pilosula* roots of maternal plant (MR). (B) GC-MS chromatogram of the *C. pilosula* roots of *in vitro* regenerated plants (IR).

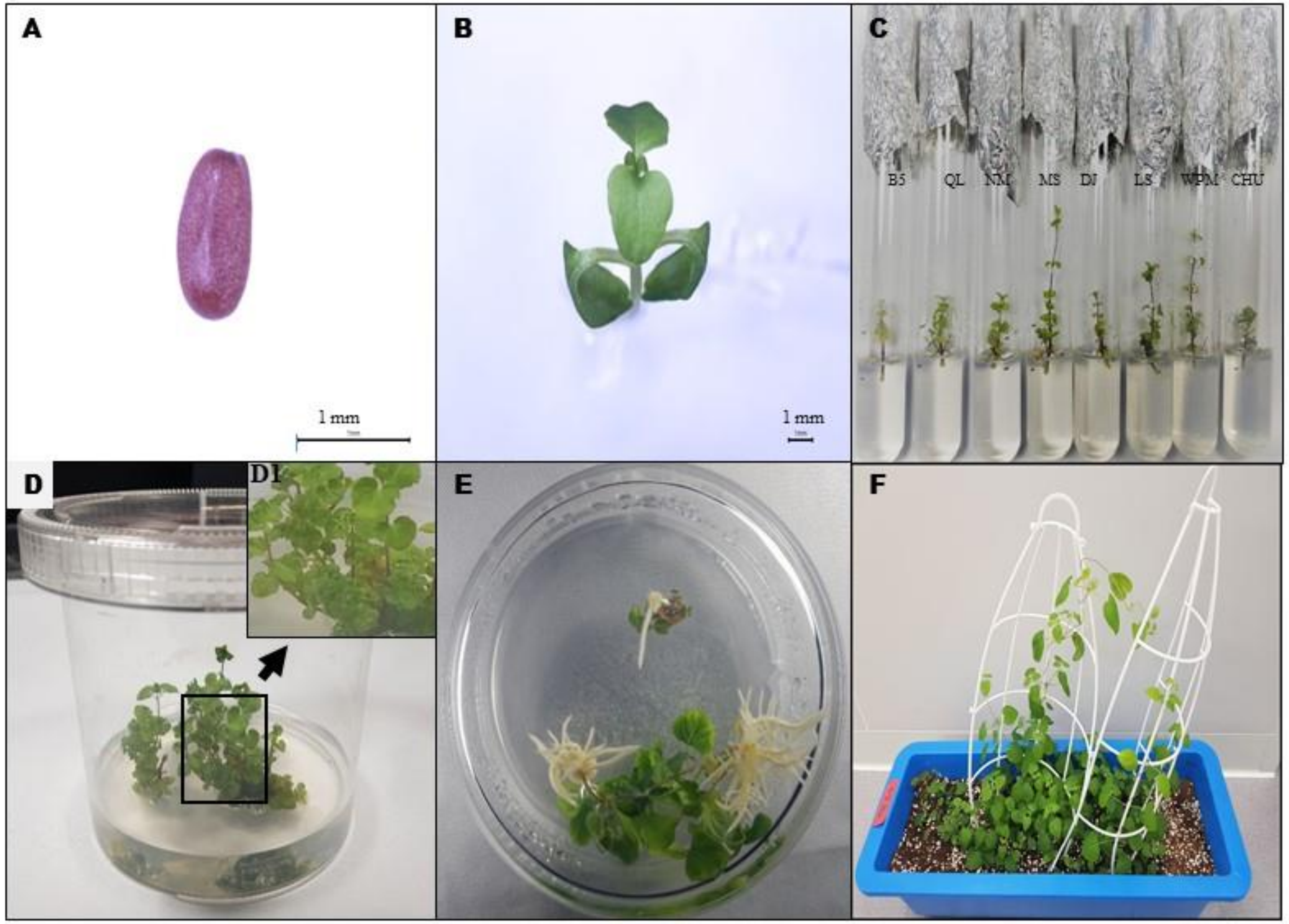

Supplementary Fig.3 *C. pilosula in vitro* propagation through apical shoot in optimum growth conditions. (A) Seed of *C. pilosula*. (B) *In vitro C. pilosula* seedling for use as explants. (C) Media test for growth of *C. Pilosula* (D) Test of different cytokines for shoot proliferation of *C. Pilosula*. (D1) Multiple shoots developing from a single apical shoot. (E) Test for rooting of *C. pilosula* in different Auxins. (F) Acclimatized regenerated *C. pilosula* plant with well-developed root and shoot systems in horticulture soil mixed with perlite in the ratio of 2:1.

Supplementary Table 1 List of primers, their sequences, number of scorable bands and approximate sizes of the amplified fragments generated by the 12 RAPD markers.

No.	Primer code	Primer Sequences (5'-3')	Number of scorable bands	Approximate range of amplification (bp)
1	OPA-13	CAGCACCCAC	5	500 ~ 1500
2	OPB-09	TGGGGGACTC	1	1500 ~ 2000
3	OPE-14	TGCGGCTGAG	4	800 ~ 3000
4	OPF-01	ACGGATCCTG	2	1000 ~ 1500
5	OPG-03	GAGCCCTCCA	4	700 ~ 2000
6	OPI-16	TCTCCGCCCT	1	700 ~ 800
7	OPA-02	TGCCGAGCTG	2	800 ~ 1000
8	OPB-01	GTTTCGCTCC	2	800 ~ 1500
9	OPC-15	GACGGATCAG	4	700 ~ 3000
10	OPE-02	GGTGCGGGAA	1	1000 ~ 1500
11	OPA-16	AGCCAGCGAA	1	800 ~ 900
12	OPB-17	AGGGAACGAG	2	500 ~ 2000
			**Total 29**	**Average 816-1725**
